# Did RNA editing in plant organellar genomes originate under natural selection or through genetic drift?

**DOI:** 10.1186/1745-6150-3-43

**Published:** 2008-10-21

**Authors:** Richard W Jobson, Yin-Long Qiu

**Affiliations:** 1 Department of Ecology and Evolutionary Biology, University of Michigan, Ann Arbor, Michigan 48109-1048, USA; 2 Centre Nationale de la Recherche Scientifique, Institut des Sciences de l'Evolution, CC064, Université Montpellier 2, Place Eugène Bataillon, 34095 Montpellier Cedex 05, France

## Abstract

**Background:**

The C↔U substitution types of RNA editing have been observed frequently in organellar genomes of land plants. Although various attempts have been made to explain why such a seemingly inefficient genetic mechanism would have evolved, no satisfactory explanation exists in our view. In this study, we examined editing patterns in chloroplast genomes of the hornwort *Anthoceros formosae *and the fern *Adiantum capillus-veneris *and in mitochondrial genomes of the angiosperms *Arabidopsis thaliana*, *Beta vulgaris *and *Oryza sativa*, to gain an understanding of the question of how RNA editing originated.

**Results:**

We found that 1) most editing sites were distributed at the 2^nd ^and 1^st ^codon positions, 2) editing affected codons that resulted in larger hydrophobicity and molecular size changes much more frequently than those with little change involved, 3) editing uniformly increased protein hydrophobicity, 4) editing occurred more frequently in ancestrally T-rich sequences, which were more abundant in genes encoding membrane-bound proteins with many hydrophobic amino acids than in genes encoding soluble proteins, and 5) editing occurred most often in genes found to be under strong selective constraint.

**Conclusion:**

These analyses show that editing mostly affects functionally important and evolutionarily conserved codon positions, codons and genes encoding membrane-bound proteins. In particular, abundance of RNA editing in plant organellar genomes may be associated with disproportionately large percentages of genes in these two genomes that encode membrane-bound proteins, which are rich in hydrophobic amino acids and selectively constrained. These data support a hypothesis that natural selection imposed by protein functional constraints has contributed to selective fixation of certain editing sites and maintenance of the editing activity in plant organelles over a period of more than four hundred millions years. The retention of genes encoding RNA editing activity may be driven by forces that shape nucleotide composition equilibrium in two organellar genomes of these plants. Nevertheless, the causes of lineage-specific occurrence of a large portion of RNA editing sites remain to be determined.

**Reviewers:**

This article was reviewed by Michael Gray (nominated by Laurence Hurst), Kirsten Krause (nominated by Martin Lercher), and Jeffery Mower (nominated by David Ardell).

## Background

RNA editing refers to the general phenomenon of post-transcriptional modification of gene transcripts, including insertion, deletion and substitution of nucleotides [1,2]. In plants, substitution types of RNA editing, mostly C to U but occasionally U to C, have been observed in both chloroplast and mitochondrial genomes of all major lineages of land plants [3-15].

Previous studies have shown genome-wide occurrence of RNA editing in both chloroplasts and mitochondria [8,10-15], restoration of conserved amino acids by RNA editing [3-5,11,13,15-17], increases of protein hydrophobicity after editing [8,11], correlation of editing frequency with GC content in a gene [18], possible association of RNA editing with gene regulation [8,19], and gain and loss of edit sites through time [20]. The phylogenetic distribution of RNA editing across lineages of plants has also been investigated, with patterns of editing site gain and loss found to be mostly lineage specific [7,9,12,21-23].

Despites these studies, the fundamental question of how RNA editing originated in plant organellar genomes remains largely unanswered. Some researchers have suggested a hypothesis of genetic drift resulting in both the appearance of editing activity and fixation of mutations at editable sites [24-26], with a possibility of natural selection maintaining RNA editing activity [24]. However, there has been no analysis using empirical data to explicitly test this hypothesis. In particular, no comprehensive analysis has been performed to examine the extent of functional difference resulted from amino acid changes caused by RNA editing, even though it is generally recognized that RNA editing involves functionally important changes in both protein-coding and structural and regulatory RNA-coding sequences. Further, in spite of the observation of hydrophobicity difference of amino acid changes involved in RNA editing in *Arabidopsis thaliana *and *Anthoceros formosae *[8,11], no attempt has been made to evaluate this change of an important physico-chemical property of amino acids from a broad perspective of the cellular compartment environment in both organelles. Chloroplasts and mitochondria house disproportionately large percentages of genes encoding membrane-bound proteins involved in electron transport and ATP synthesis in energy metabolism [27]. Finally, the genomic and sequence environment in which RNA editing arose remains poorly known.

Over the last ten years, a number of plants have been investigated for genome-wide RNA editing by comparing the fully sequenced organellar genomes with their mature transcriptomes. These include 11 diverse land plants for the chloroplast genome: the hornwort *Anthoceros formosae *[11,17], the fern *Adiantum capillus-veneris *[13,28], the gymnosperm *Pinus thunbergii *[29,30], and the angiosperms *Cuscuta reflexa *and *C. gronovii *[15], *Arabidopsis thaliana *[31,32], *Nicotiana tabacum *[33,34], *Atropa belladonna *[35], *Phalaenopsis aphrodite *[36], *Saccharum officinarum *[37], and *Zea mays *[38]. For the mitochondrial genome, five angiosperms have been investigated: *A. thaliana *[8,39], *Brassica napus *[10], *Beta vulgaris *[14,40], *Oryza sativa *[41], and partially for *Zea mays *[42].

In the *A. formosae *chloroplast genome there are 509 C→U sites distributed across 68 genes and eight ORF's, with 94, 397 and 12 sites at 1^st^, 2^nd^, and 3^rd ^codon positions respectively. A majority of editing events involves codon changes from NCN to NUN (Ser, Pro, Thr, Ala → Phe, Leu, Ile, Met, Val) with editing at 2^nd ^positions. The 433 U→C sites in this genome are distributed across 69 genes, with 264, 149, and 16 sites occurring at 1^st^, 2^nd^, and 3^rd ^codon positions respectively. 81% of the edited 1^st ^positions convert Stop (UAR) codons to those of Gln (CAR), and Cys/Stop/Trp (UGN) codons to those of Arg (CGN) [11,17].

In the *A. capillus-veneris *chloroplast genome, 51 C→U, and all 35 U→C editing events occur in 1^st ^positions, while 226 and 20 C→U changes are found at 2^nd ^and 3^rd ^positions respectively. Of these editing events 75% of the 1^st ^position U→C edits consist of Stop (UGA) to Arg (CGA) codon changes. The C→U changes are found across 52 genes, while U→C editing events occur in 20 genes [13].

In comparison, the chloroplast genomes of seed plants contain only ~15–44 C→U editing sites, distributed across ~15 genes [15,22,26,29-38]. There has been an apparent loss of RNA editing sites in the chloroplast genome during evolution of land plants [20,26].

While the mitochondrial genome of *Marchantia polymorpha *(a liverwort) lacks RNA editing [9,43], a large number of editing sites have been found in three angiosperm mitochondrial genomes. In *A. thaliana*, 441 C→U sites are spread across 30 genes with the majority found at the 2^nd ^codon positions (236), followed by 1^st ^(154), and 3^rd ^(51) positions. 57% of 1^st ^position editing involves URN to CRN changes [8]. For *B. vulgaris *353 C→U sites were identified across 30 protein coding genes with 125, 198, and 30 sites found at 1^st^, 2^nd^, and 3^rd ^codon positions respectively [14]. In *O. sativa*, a similar pattern has been observed, with 153, 246, and 38 editing sites at 1^st^, 2^nd^, and 3^rd ^positions of 29 genes respectively [41].

These data provide an opportunity to take a comparative genomic approach to investigate the question of how RNA editing evolved in plant organellar genomes. In this study, we analyzed the sequence and editing data from fully characterized organellar genomes of *A. formosae, A. capillus-veneris, A. thaliana, B. vulgaris*, and *O. sativa*, to gain a better understanding of conditions under which RNA editing evolved. Since *B. napus *and *Z. mays *are closely related to *A. thaliana *and *O. sativa *respectively, and their editing patterns are similar to each other, they were excluded from our analyses. The chloroplast genomes of *P. thunbergii, A. thaliana*, *N. tabacum*, *A. belladonna*, *Z. mays*, and other angiosperms were not analyzed here because they contained only a small number of editing sites and might not be suitable for detecting general patterns.

We first examined types of codons that were edited and their editing frequencies. In particular, we investigated the relationship between the change in hydrophobicity and molecular size of amino acids encoded by pre- and post-edited codons and the editing frequency. This analysis should allow us to assess functional significance of RNA editing. In the second set of analyses, we calculated frequencies of 20 amino acids in two groups of genes that encode membrane-bound and soluble proteins respectively, in both organelles. Comparison of occurrence frequencies of amino acids in the two organelles with the editing frequencies of codons could help identify cellular environmental factors that might have contributed to and shaped the origin and evolution of RNA editing. Finally, we performed correlation analyses between editing frequencies and i) nucleotide frequencies at the 1^st^, 2^nd^, 3^rd^, and total codon positions in all genes, and ii) gene specific rates of evolution at both synonymous (*d*_*S*_) and nonsynonymous (*d*_*N*_) sites. These analyses might help to reveal the sequence environment in which editing evolved.

## Materials and methods

### Taxon sampling and annotation of editing sites

The genome sequences used in this study were obtained from the GenBank organellar genome database .

**Figure 1:**
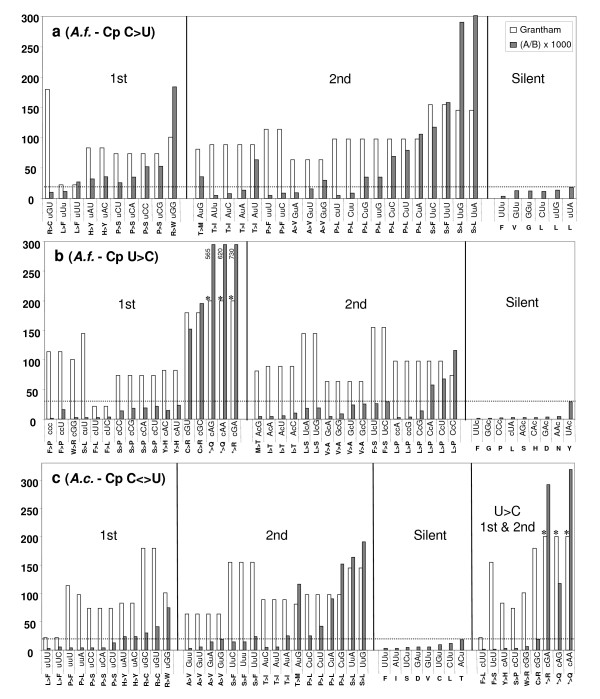
**Editing frequency {(A [number of edited codons]/B [number of codons in the genome]) × 1000} versus Grantham index**[** 47 **]**for all edited codons in protein coding genes in chloroplasts of *****A. formosae *****(a, forward editing; b, reverse editing), and***** A. capillus-veneris *****(c, forward and reverse editing). Amino acid residues are shown as pre-edited to edited below all codons.** The vertical axis indicates Grantham index value and editing frequency. Edited codons are grouped into nonsynonymous 1^st^, 2^nd^, and silent site changes. Asterisks indicate arbitrarily assigned values of 200 for Grantham index values in premature stop codons. The dotted line denotes an upper limit of editing frequency across silent editing sites. The values obtained for A/B were multiplied by 1000 to make these data plottable against Grantham's values.

**Figure 2:**
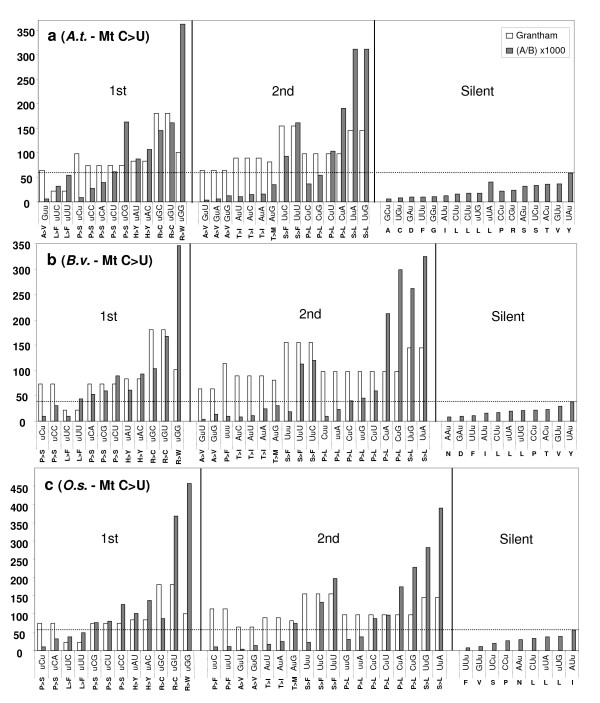
**Editing frequency {(A [number of edited codons]/B [number of codons in the genome]) × 1000} versus Grantham index**[** 47 **]**for all edited codons in protein coding genes in mitochondria of *****A. thaliana *****(a),***** B. vulgaris *****(b), and***** O. sativa *****(c). Amino acid residues are shown as pre-edited to edited below all codons.** The vertical axis indicates Grantham index value and editing frequency. Edited codons are grouped into nonsynonymous 1^st^, 2^nd^, and silent site changes. The dotted line denotes an upper limit of editing frequency across silent editing sites. The values obtained for A/B were multiplied by 1000 to make these data plottable against Grantham's values.

**Figure 3:**
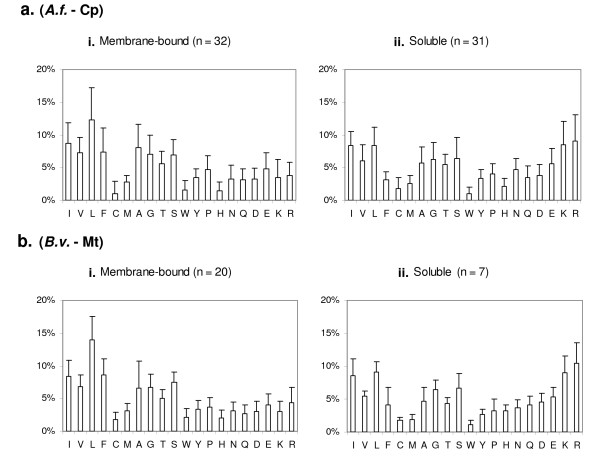
**Frequencies of amino acids in membrane-bound and soluble proteins in the chloroplast of *A. formosae *(a) and the mitochondrion of *B. vulgaris *(b).** The "n" indicates the number of genes in each class. Plots i contain membrane-bound protein genes. Plots ii contain genes encoding soluble proteins. Amino acid residues are ordered from the most hydrophobic (left) to the most hydrophilic (right) according to the Kyte-Doolittle hydrophobicity scale [48].

Annotation of RNA editing sites was performed for individual gene sequences in accordance with editing sites determined by original authors from cDNA sequences across 68 chloroplast genes of *Anthoceros formosae *(GenBank accession no. NC_004543) [11,17] and *Adiantum capillus-veneris *(GenBank accession no. AY178864) [13,28]. For *A. formosae*, 11 genes were determined not to contain either forward or reverse editing sites, while for *A. capillus-veneris *forward and reverse editing sites were not present in 16 and 49 genes respectively. The same procedure was carried out for mitochondrial genes of *Arabidopsis thaliana *(GenBank accession no. NC_001284) [8,39],*Beta vulgaris *(GenBank accession nos. NC_002511, DQ381444-DQ381465) [20,40], and *Oryza sativa *(GenBank accession no. BA000029) [41]. For *A. thaliana*, *B. vulgaris*, and *O. sativa*, 30 of 33, 30 of 31, and 28 of 29 analyzed genes contained C→U editing sites respectively (U→C editing was not observed in these three genomes). Compositional data for codons (Figures 1, 2), and amino acids (Figure 3), were obtained from the relevant abovementioned genomes.

### Multi-gene correlations of T-A and G-C distance versus editing frequency

**Figure 4:**
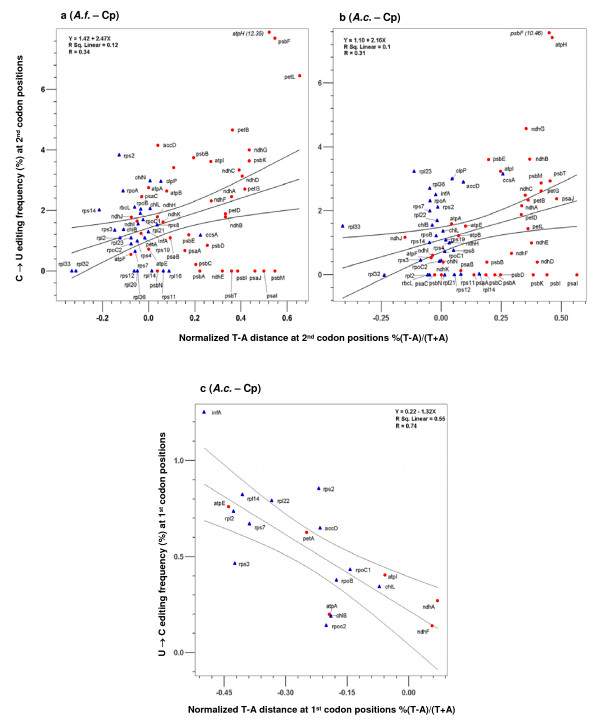
**The correlation of editing frequency and T-A distance at the 2^nd ^and 1^st ^codon positions in chloroplast genomes of *A. formosae *(a) and *A. capillus-veneris *(b, c).** Membrane-bound protein coding genes are indicated by red dots, while soluble protein coding genes are presented as blue triangles. Regression results are significant for all presented analyses (p < 0.01) with details shown in each figure. Values in brackets indicate data points not included in the analyses due to excessive deviation from the mean (> 3 standard deviations). For clarity, the data of Figure 4c were re-analyzed using only the 19 genes containing reverse editing. However, the significant result obtained using all genes are presented in Additional file 1.

**Figure 5:**
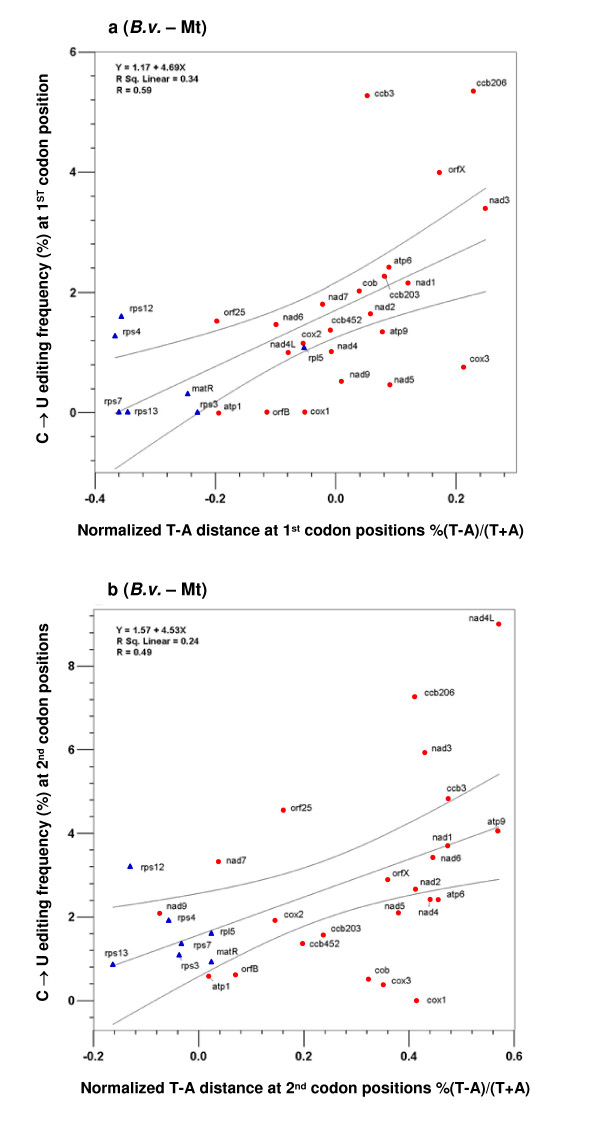
**The correlation of editing frequency and T-A distance at the 2^nd ^and 1^st ^codon positions in the mitochondrial genome of *B. vulgaris *(a, b).** Membrane-bound protein coding genes are indicated by red dots, while soluble protein coding genes are presented as blue triangles. Regression results are significant for all presented analyses (p < 0.01) with details shown in each figure.

To examine possible correlation of nucleotide composition with RNA editing across genes of *A. formosae*, *A. capillus-veneris*, *A. thaliana*, *B. vulgaris*, and *O. sativa*, 2-tailed Pearson correlation analyses were conducted. We calculated the RNA editing frequency (%) per gene as ((number of edited sites/gene length) × 100) for first, second, third, and total codon positions. The ratio of compositional difference between nucleotide pairs thymidine and adenosine (T-A), and cytidine and guanosine (C-G), at first, second, third, and total codon positions, was calculated as %(T - A)/(T + A), and %(C - G)/(C + G). Significant associations (α < 0.01) for these two variables were determined through linear regression analysis (Figures 4, 5, Additional file 1). For analysis of RNA editing frequency (%) versus T-A distance in 2^nd ^codon positions, one extreme value was present in each data set (Figure 4a, b). These values were found to be > 3 standard deviations from the mean (diagnosed using the Cook's Distance procedure) [44], and were omitted from the analysis.

### Multi-gene correlations of rates of evolution versus editing frequency

Gene specific synonymous (*d*_*S*_) and nonsynonymous (*d*_*N*_) rates were estimated using CODONML implement in PAML [45]. The underlying model was M1+F3X4 [45]. Data sets were made of 11 and 7 taxa, across 53 and 21 edited orthologous genes, representing the conserved chloroplast and mitochondrial proteomes respectively (Additional file 2). Branching order of taxa followed the phylogenetic hypothesis of [46]. Genes were omitted from the analysis if they were found to be absent in two or more of the candidate genomes, or if multiple alignment was spurious. The *d*_*N*_*/d*_*S *_ratio, *d*_*N*_, and *d*_*S *_were the summed estimates across all branches of the tree (Additional file 2).

## Results and discussion

### All heavily edited codons encode amino acids with significantly different hydrophobicity and molecular size than pre-edited codons, and editing greatly increases the hydrophobicity of proteins

Hydrophobicity and molecular size are two fundamental physico-chemical properties of amino acids that determine protein structure and functionality, and naturally they greatly influence amino acid replacement rate during protein evolution [47]. In our analyses of editing frequencies of codons and the corresponding amino acid changes involved, we found that the changes of Ser → Leu codons represent the highest class of conversions for the 2^nd ^codon position editing in both chloroplasts and mitochondria, and that the changes of Ser → Phe codons and Pro → Leu codons take the next two places in ranking by editing frequency.

Together, these three classes account for 73–98% of the editing events at the 2^nd ^codon positions and 49–68% of all editing events in the two organelles (Figures 1, 2). For the 1^st ^codon position editing, the changes of Arg → Trp codons always takes the lead, with those of Arg → Cys codons, Pro → Ser codons, and His → Tyr codons accounting for much of the remaining editing events, again in both organelles. All these amino acid changes involve large differences of hydrophobicity and molecular size as measured by the Grantham index [47] and the Kyte-Doolittle hydrophobicity scale [48].

In addition, a similar pattern is observed across the ~15–44 C→U editing sites found in the chloroplast genomes of seed plants [15,22,26,30-37].

For reverse editing in chloroplasts of both *A. formosae *and *A. capillus-veneris*, the highest class of conversions involves three stop codons, which are converted to Arg or Gln codons after editing at 1^st ^codon positions, and they represent 60% and 77% of all reverse editing events at this codon position. The significance of this class of editing on protein functionality is self-evident. The changes of Cys → Arg codons and Leu → Pro codons represent most remaining editing events at 1^st ^and 2^nd ^codon positions, respectively. There are a few other classes of codon changes resulted from editing that also involve changes of functionally very different amino acids, but they occur at much lower frequencies than the ones mentioned above. Finally, there are a small number of editing events that cause changes of functionally similar amino acids or do not alter amino acid identity at all (Figures 1, 2). However, we found no case of heavy editing for codon substitutions that are translationally silent or involved little change of the physico-chemical properties of amino acids.

These data clearly and consistently suggest that RNA editing plays an important functional role in minimizing effect of mutations that would have greatly altered protein structure through amino acid replacement involving drastic changes in hydrophobicity and molecular size. Earlier studies have reported restoration of conserved amino acids by editing [3-5,11,13,15,16], but the patterns, and moreover the consistency of these patterns, in both chloroplast and mitochondrial genomes of such diverse land plants shown here are really striking, and they are based on much more extensive data than those earlier studies.

Previously, editing in transcripts of tRNA genes, putative Shine-Dalgarno sequences, and group II introns has also been shown to improve structural folding or base-pairing of those molecules [8,11,13,49,50].

Recently, Mower and Palmer [14] also found that in the mitochondrial genome of *B. vulgaris*, partial editing was much more widespread at silent sites (58.5%) than non-silent sites (8.0%), a phenomenon that was reported earlier on single genes [51,52]. Even though partial editing may represent editing in progress in intermediate reaction products [53] the discrepancy in silent and non-silent sites is more consistent with a functional explanation. Therefore, these data and observations from previous studies unambiguously support a role of natural selection in maintaining RNA editing activity in plant organelles [24].

Another observation we made from these data is that in chloroplasts or mitochondria of these five plants, editing uniformly increases protein hydrophobicity when we examined amino acid changes of editing events using the Kyte-Doolittle hydropathy scale of amino acids [48]. Even in *A. formosae *where almost an equal number of forward and reverse editing events were reported, this increase of protein hydrophobicity still holds because a large proportion of reverse editing events were involved in resurrecting internal stop codons and did not offset the hydrophobicity increase generated by forward editing events (Figure 1a, b). This phenomenon has been observed earlier in mitochondria of *A. thaliana *[8] and in chloroplasts of *A. formosae *[11]. Now it seems reasonable to suggest that the phenomenon is more widespread in land plants than previously known and may apply to most RNA editing events in organelles of most land plants. If this is the case, one might wonder whether there is any functional reason behind this pattern of amino acid changes mediated by RNA editing, which may lead closer to the answer to the puzzle of origin of RNA editing in plant organelles.

### Plant organellar genomes contain disproportionately large percentages of genes encoding membrane-bound proteins rich in hydrophobic amino acids

After their endosymbiotic origins from cyanobacteria and proteobacteria, chloroplasts and mitochondria became organelles in eukaryotic cells specializing in energy metabolism. They both contain two large sets of genes, one set encoding proteins for either photosynthesis or aerobic respiration and the other set encoding proteins for information processing, namely, gene transcription, splicing and translation [27,54,55].

While proteins involved in information processing, Calvin cycle in chloroplasts and Krebs cycle in mitochondria are soluble ones located in stroma or matrix (actually most of the genes encoding these proteins were already transferred to the nucleus after endosymbioses), those involved in photon capture (photosystem proteins) and photophosphorylation (electron carriers and ATPase) in chloroplasts and oxidative phosphorylation (electron carriers and ATPase) in mitochondria are all embedded in phospholipid bilayer membranes. Hence, it is clear that these two organelles house much larger percentages of genes encoding membrane-bound proteins than their free-living bacterial ancestors or the nucleus of the eukaryotic cell [27,54,55].

When we examined occurrence frequencies of 20 amino acids in two classes of proteins encoded by chloroplast and mitochondrial genomes of *A. formosae *and *B. vulgaris*, respectively, we found that hydrophobic amino acids such as Leu, Ile, Phe, Val, Gly, Ser and Ala were used much more frequently than hydrophilic ones like His, Trp, Arg and Lys in the membrane-bound proteins (Figure 3). Leu consistently ranked the highest among all 20 amino acids in membrane-bound proteins of both organelles. In soluble proteins, no such pronounced bias in amino acid usage was observed, and instead hydrophilic amino acids such as Arg and Lys were used as much as hydrophobic ones like Leu and Ile (Figure 3). These data fit basic physical chemistry expectation in that abundance of membrane-bound proteins in chloroplasts and mitochondria naturally dictates frequent use of hydrophobic amino acids in synthesizing these proteins. Since gene contents in both organellar genomes have been evolutionarily conserved throughout land plants, the above pattern of amino acid usage observed from the two species can be assumed to represent the general pattern of amino acid usage in both organelles of all land plants.

Examination of the genetic code table shows that all codons with U at the 2^nd ^positions encode hydrophobic amino acids: Leu, Ile, Phe, Val, and Met. The fact that Leu has the highest frequency of occurrence in both organelle-encoded proteomes shown above (Figure 3) and is encoded by six codons all with U at the 2^nd ^positions can now explain why Ser → Leu and Pro → Leu codon changes represent the two most frequent classes of editing events observed in our study (Figures 1, 2). These data unequivocally demonstrate the functional basis of RNA editing in plant organellar genomes.

### RNA editing sites occur more frequently in ancestrally T-rich sequences, which are more abundant in genes encoding membrane-bound proteins with many hydrophobic amino acids

To understand the sequence environment in which RNA editing sites evolved, we investigated correlation between editing frequency and T-A and C-G distance across different genes. The latter measures the amount of excess of T over A, and C over G, at certain codon positions, under the assumption that DNA sequences in a genome evolve towards T = A and C = G in line with the 'second parity rule' [56]. For analyses of mitochondrial genes in *A. thaliana*, *B. vulgaris*, and *O. sativa *we present only the results *B. vulgaris *as correlations were very similar in magnitude and distribution (Figure 5; Additional file 1).

At the 2^nd ^codon positions, there was a positive correlation between these two parameters in the three genomes we examined: chloroplast genomes of *A. formosae *and *A. capillus-veneris *and the mitochondrial genome of *B. vulgaris *(Figures 4a, b, 5b). In other words, in genes that showed larger T-A distances or had an excess of T over A in their sequences, there were more editing events. This positive correlation between editing frequency and T-A distance was also seen at the 1^st ^codon positions in the mitochondrial genome (Figure 5a). As expected, a significant negative correlation between the frequency of reverse editing and T-A distance was found at 1^st ^codon positions in the chloroplast genome of *A. capillus-veneris *(Figure 4c), but not in that of *A. formosae *(Additional file 1). These findings are not unexpected, as when there was abundance of T in a gene, one would expect more T→C mutations, which would then be fixed and later experience C→U changes during editing because of existence of the editing machinery. Interestingly, T-A distance for 3^rd ^codon positions did not show any correlation across all of the editing frequency comparisons (Additional file 1).

The other observation we made from these data was that the genes encoding membrane-bound proteins tend to have larger T-A distances in their sequences and thus more editing events (Figures 4, 5). All the genes that are at high ends of the correlation plots encode membrane-bound proteins, whereas most of those located at low ends of the plots encode soluble proteins. These data are again consistent with the idea we formulated above that the high proportion of membrane-bound proteins in plant organelle-encoded proteomes seems to be responsible for the prevalence of RNA editing in these two genomes.

In these correlation analyses, the sequences of mature transcripts were used, which was based on an implicit assumption that the edited sequence represented an ancestral condition and the pre-edited sequence represented a derived condition. In other words, RNA editing was assumed to have evolved recently in eukaryotic evolution. Many previous studies have indeed observed that editing generally restores evolutionarily conserved amino acids [3-5,11,13,15,16]. It has also been shown that only a small number of editing sites are evolutionarily conserved across land plants in the chloroplast genome [7,13,15,26]. The same is true in mitochondrial genomes of *A. thaliana, B. vulgaris and O. sativa*; only 118 sites (21%) of a total of 561 editing sites were shared by these three diverse angiosperms according to our examination. Thus, the assumption we used in the correlation analyses seems to be valid.

### Editing sites are found more frequently in selectively constrained genes

**Figure 6:**
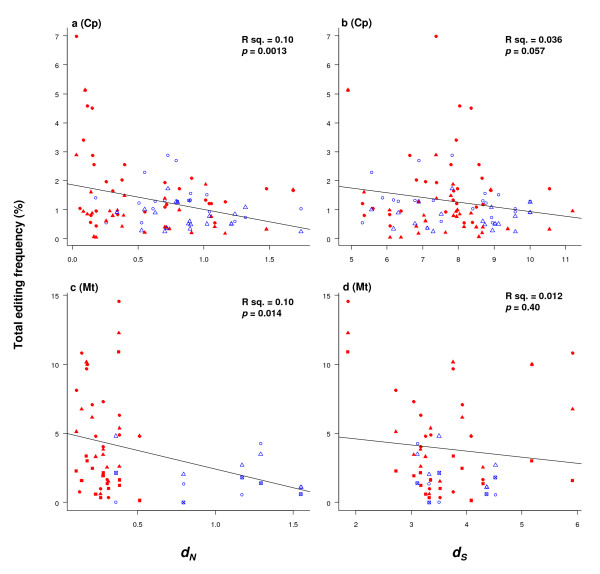
**The correlation of total editing frequency and gene specific rates of molecular evolution at nonsynonymous (*d*_*N*_) (a, c) and synonymous (*d*_*S*_) (b, d) sites in chloroplast genomes of *A. formosae *and *A. capillus-veneris *(a, b), and mitochondrial genomes of *A. thaliana*, *B. vulgaris*, and *O. sativa *(c, d).** Solid points (red) signify membrane-bound proteins, while open points (blue) represent soluble proteins. Data for *A. formosae *are shown as triangles, while *A. capillus-veneris *is presented as circles (a, b). Data for *A. thaliana*, *B. vulgaris*, and *O. sativa *are presented as squares, triangles, and circles respectively (c, d).

Because of the clear functional basis of RNA editing demonstrated above, we suspected that RNA editing would increase in line with the selection constraint on genes. We then examined possible correlation of gene specific rates of evolution, at both synonymous (*d*_*S*_) and nonsynonymous (*d*_*N*_) sites, with total RNA editing frequency. These analyses were performed across genes of *A. formosae*, *A. capillus-veneris*, *A. thaliana*, *B. vulgaris*, and *O. sativa *(Figure 6). Editing frequency data included all codon positions, and chloroplast editing data combined both forward and reverse editing frequencies.

Initially, the correlations were confounded by the effect of editing sites themselves, which, when included in the estimation of molecular evolutionary rates, would invoke circularity when compared against editing frequency. In addition, frequent in-frame stop codons in many chloroplast genes interfered with the analysis. To overcome these problems we removed editing sites from the analyses by converting the sites to the ancestral bases.

The results obtained for both genomes suggest that frequently edited membrane-bound genes are under stronger selection constraint than those for soluble proteins (Figure 6a, c).

We also determined that there is little relationship between editing frequency and synonymous rates (Figure 6b, d), which is an approximation of the neutral mutation rate.

These findings corroborate the observation of reduced editing frequency in chloroplast genomes of two parasitic plants [15], in which many retained genes have undergone reduced selection pressure in two different species from the same lineages [57].

### Did RNA editing in plant organellar genomes originate under natural selection or through genetic drift?

Ever since the discoveries of RNA editing in plant organellar genomes [3-6], geneticists and molecular evolutionists have been intrigued by the question how RNA editing evolved in the first place [8,24-26]. Recently, Tillich et al. [26] uncovered a pattern termed 'preferred sequence context' in which there may be a preference for pyrimidines immediately upstream, and purines immediately downstream of an editing site. Such a pattern could be important for site recognition by the RNA editing machinery. In an earlier study, Covello and Gray [24] insightfully dissected the complex question into three parts: 1) the origin of RNA editing activity, 2) fixation of mutations at editable sites, and 3) maintenance of RNA editing activity. Further, they hypothesized that genetic drift played a key role in the first two processes and natural selection contributed to the third. Some authors hold an even more neutralistic view on the origin of RNA editing than these two authors, e.g., Lynch et al. [25]. Below we present our ideas on whether RNA editing originated under natural selection or through genetic drift based on the analyses shown above. To make the argument easier to construct according to strength of the data, we follow a reverse sequence of the three steps proposed by [24].

#### Maintenance of RNA editing activity

In agreement with [24], we think that the data are overwhelming in supporting a role of natural selection in maintaining RNA editing activity in organelles of plants, despite a poor understanding of its biochemical nature until now. Without editing, replacement of so many amino acids with very different physico-chemical properties would dramatically alter protein structures, if not rendering these proteins completely disfunctional (Figures 1, 2). The most extreme case is the chloroplast genome of *A. formosae*, where forward and reverse editing events affect more than half of the genes in the genome [11]. In addition, stop and start codons are often created or removed by editing [11,13,14]. Likewise, tRNAs, group II introns, and putative Shine-Dalgarno sequences that contain editing sites are also likely to be affected if editing activity is lost [8,11,13,49,50]. Hence, we will not elaborate on this point any further here.

#### Fixation of mutations at editable sites

This is the starting point where our view differs from that of [24] on how RNA editing evolved. If mutations at editable sites were fixed through genetic drift, one would expect their distribution patterns to be completely random when examined against codon positions, types of codons, and kinds of genes. However, our analyses provided ample data to contradict this scenario. First, we found editing sites to be predominantly distributed at 2^nd ^and 1^st ^codon positions (Figures 1, 2), as reported by previous studies [8,10,11,13-15,20] and found here to be correlated across genes for all taxa examined (Additional file 1).

Second, editing affected codons that resulted in larger hydrophobicity and molecular size changes much more frequently than those with little change involved, and heavy editing uniformly increased protein hydrophobicity (Figures 1, 2). In fact, there was a conspicuous lack of heavily edited codons that were translationally silent or involved in little change of hydrophobicity and molecular size. Finally, editing occurred more frequently in selectively constrained genes encoding membrane-bound proteins than those encoding soluble ones because these genes contained many U-rich codons encoding hydrophobic amino acids (Figures 4, 5, 6). These data all support a role of natural selection in fixing mutations at editable sites and are fundamentally incompatible with a neutralistic explanation invoking genetic drift.

One point we want to clarify is that the exact point of contention in our exposition is slightly different from that of [24]. Our argument is broader than theirs in that we argue that both fixation of editing sites and fixation of mutations at editable sites were achieved by natural selection. Theoretically, upon origin of editing activity any site with T or C could mutate to C or T (i.e., any site with T or C could fall into the category of "editable sites") since it could always revert to its original condition after editing or reverse editing. In reality, only some sites might be allowed to do so, as there might be sequence motifs involved in fixing editing sites as suggested by some authors [8,11,32].

Basically, appearance of the editing machinery relaxed selection pressure on T↔C mutations. The data on distribution of editing sites among codon positions, codons, and genes as we presented above demonstrate clearly that fixation of editing sites and fixation of mutations at editable sites followed a pattern of functional selection.

#### Origin of RNA editing activity

It has been generally recognized that origin of genes encoding enzymes involved in RNA editing represents a critical step in the origin of RNA editing [8,24]. To date, the biochemical nature of the editing machinery remains poorly characterized even though a nuclear gene encoding a protein essential for RNA editing in chloroplasts has been identified in *Arabidopsis *recently [58].

It has also been suggested that the editing machinery probably evolved in early land plants [7,9,13,26], and that the same machinery may be responsible for editing in both organelles [7,9]. The fundamental question in the whole story of evolution of RNA editing is whether origin of the editing machinery was merely a historic accident or it represented an evolutionary innovation of necessity. Genetic drift was invoked for this event [24] in the face of lack of extensive data and any obvious functional explanation. With much more data and extensive analyses, we are inclined to take a different view here.

While we agree that the origin of RNA editing activity itself is clearly a neutral event, we suggest that it may confer some fitness to the organellar genomes. Otherwise, it is inconceivable that such a mutation would have been fixed and then maintained in land plant organelle genomes over a course of evolution of more than four hundred million years. We offer a hypothesis below.

One basic fact we want to point out is that editing allows a higher GC content in the genome, because forward editing permits fixation of C in the sense strand and G in the antisense strand, and genes in organellar genomes are distributed on both strands of a circular molecule. Perhaps there is an upper limit of AT% that is permitted in a genome and this upper limit may have promoted fixation of the genes encoding enzymes involved in RNA editing.

**Figure 7:**
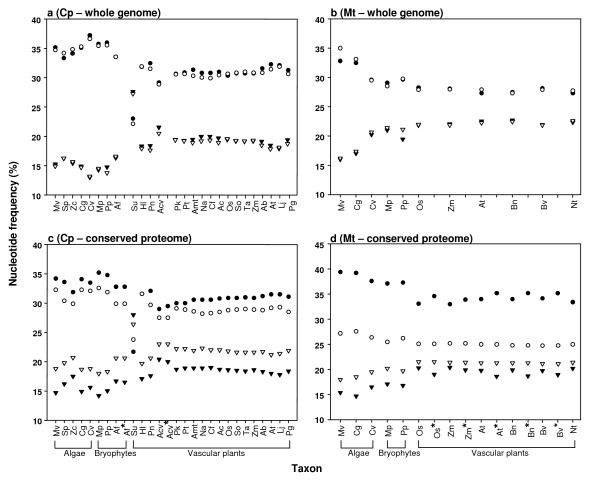
**Nucleotide frequencies across streptophytes for whole genomes of chloroplasts (a) and mitochondria (b), and combined codon positions for the conserved proteomes of chloroplasts (c) (57 genes) and mitochondria (d) (13 genes).** Nucleotides are represented by filled circles for T, open circles for A, open triangles for G, and filled triangles for C. The convergence of nucleotide composition can be observed across whole genomes and conserved proteomes. Taxa with available RNA editing data are duplicated and designated as "edit-repaired" by an asterisk. Taxa are arranged according to a phylogenetic sequence of [46] and divergence times are not considered. Broad taxonomic groupings are indicated on the X-axis below brackets. Taxonomic abbreviations are described in Additional file 3.

To strengthen this argument, we remind readers that the genetic code table and start and stop codons in this table mandate presence of all four nucleotides in a genome. Thus, AT content in any genome certainly cannot exceed 100%, and no genome consisted of less than all four nucleotides has ever been reported. Still, we acknowledge that a link between the base composition of a genome and RNA editing frequency is weak even though the two phenomena are compatible. Further, reverse editing, which occurs frequently in the chloroplast genome of *A. formosae*, and is correlated strongly with the frequency of forward editing (Additional file 1), poses a problem to our explanation. However, if organellar genomes were under pressure, not only to reduce AT, but also to equilibrate the overall nucleotide composition under the second parity rule [56], then a reduction of T would also reduce A, and an increase in C would likewise increase G. Figure 7 shows the dramatic convergence of nucleotide composition toward the equilibrium value (25%) during the early evolution of land plants. This pattern is observed both at whole genome level and across conserved protein-coding genes in both mitochondria and chloroplasts, and may signify a 'drive' for convergence of the four nucleotides toward the equilibrium (Figure 7, Additional file 3). One extreme example of nucleotide convergence can be observed in the chloroplast genome of the lycophyte *Selaginella uncinata *(Figure 7a, c) [59], in which C→U editing affects ~5% of the total coding region (S. Tsuji, pers. comm.).

The most difficult part of the RNA editing story to explain is the lineage-specific occurrence of a good number of editing sites, as many of them seem to have evolved rather recently [7, 13, 26, this study]. Colonization of land by charophytic algal ancestors of land plants, life history characteristics of individual species, the evolutionary history of particular lineages, lineage-specific GC contents in organellar genomes, different substitution rates in chloroplast and mitochondrial genomes of some plants, different degrees of asymmetrical distribution of genes on two strands in the circular organellar genomes, DNA polymerase infidelity and dNTP pool imbalances, and varying intragenomic rearrangement rates in different plant lineages may have all or partly contributed to the origin of editing activity. Especially, the first three processes could result in species undergoing bottleneck periods during which genetic drift could play a large role in fixing random mutations. However, explicit data are needed to tie any of these factors to evolution of RNA editing before a hypothesis favoring any one of them can be seriously entertained.

## Conclusion

Our analyses of RNA editing patterns in chloroplast genomes of the hornwort *A. formosae *and the fern *A. capillus-veneris *and mitochondrial genomes of three angiosperms, *A. thaliana, B. vulgaris *and *O. sativa *show that editing mostly affects functionally important and evolutionarily conserved codon positions, codons and genes encoding membrane-bound proteins. In particular, occurrence of abundant RNA editing events in plant organellar genomes may be attributed to the disproportionately large percentages of genes in these two genomes that encode membrane-bound proteins, which are rich in hydrophobic amino acids encoded by the 2^nd ^position U (T)-rich codons. These data support a hypothesis that natural selection imposed by protein functional constraints has contributed to selective fixation of certain editing sites and maintenance of the editing activity in plant organelles over a period of more than four hundred millions years. The retention of genes encoding RNA editing activity may be driven by forces that shape nucleotide composition equilibrium in two organellar genomes of these plants. However, the causes of lineage-specific occurrence of a large portion of RNA editing sites remain to be determined.

## Abbreviations

A: adenosine; C: cytidine; cDNA: complementary DNA; cp: chloroplast; G: guanosine; mt: mitochondrion; T: thymidine; U: uridine

## Competing interests

The authors declare that they have no competing interests.

## Authors' contributions

RWJ and YLQ designed and performed research, analyzed data, and wrote the paper.

## Reviewer comments

### Reviewers report 1: Michael Gray, Department of Biochemistry and Molecular Biology, Dalhousie University

In the original three-step model of Covello and Gray (On the evolution of RNA editing. *Trends Genet*. 1993 Aug;9(8):265-8), it is admittedly rather difficult to cleanly differentiate among the three steps because the model is so obviously a co-evolutionary one (editing sites + editing machinery). A salient point that Jobson and Qiu make here is that "the genomic and sequence environment in which RNA editing arose remains poorly known". That's an important point because it somewhat constrains what we can infer about the beginnings of plant RNA editing by looking at the end result (i.e., contemporary organellar genomes).

#### Authors' response

*The original three-step model of origin and evolution of RNA editing proposed by Covello-Gray (1993) is still a logical one in our opinion. That is why we used it to construct the last part of our discussion in the paper in light of the new data. We disagree with the co-evolution argument. The RNA editing activity (or machinery) has to evolve first; otherwise editing sites will not appear because mutations at those sites will almost certainly be wiped out and not be fixed given the drastic phenotypic effect they cause as demonstrated in this paper*.

It also matters precisely what one means by 'originate', and where the transition from 'origin' to 'maintenance' occurs. Jobson and Qiu agree, as set out in the Covello-Gray model, that "the origin of RNA editing activity itself is clearly a neutral event", although they suggest that "it may confer some fitness to the organellar genomes". I wouldn't dispute the latter statement, especially if RNA editing originated in a relatively AT-rich genome whose descendents were increasingly subjected to a GC drive that tended to make the genome more GC-rich. The hydrophobicity argument the authors make is an excellent one, so that RNA editing would not only have the consequence of 'correcting' obvious 'mistakes' (such as the edits that serve to generate two essential ligands in the Cu_A _binding site of wheat Cox2, or creation of translation start and/or stop signals) but a more subtle function of ensuring that the amino acid composition of predominantly membrane-associated proteins is optimized. Even so, it's not clear to me that Jobson and Qiu aren't already talking about selective forces operating at the level of maintenance, rather than at the level of origin per se. As to whether the origin of the editing machinery "was merely a historic accident or it represented an evolutionary innovation of necessity", a primary tenet in evolutionary theory is that evolution cannot be and is not anticipatory, so that I would find 'innovation of necessity' a difficult concept to swallow. However, once having arisen in a neutral context, such an activity would most certainly be subject to strong selection, for all the reasons the authors enunciate. Thus, Jobson and Qiu list a number of factors that they suggest could have contributed to the *origin *of editing activity. In contrast, I would argue that these factors would have contributed to the *maintenance *of editing activity (i.e., selection). This view would address the authors' concern about how "such machinery would have been maintained in land plant organelles over a course of evolution of more than four hundred million years."

[On the issue of RNA editing machinery, we have to consider not only the catalytic activity (deaminase(?), at least for C→U) but also the many trans-acting protein factors (PPR proteins?) that evidently provide the specificity for plant organellar editing. The evolution via multiple gene duplications and diversifications of the interesting multi-gene family that encodes PPR proteins may provide clues as to such features as the lineage-specific editing on which Jobson and Qiu comment.

#### Authors' response

*Although we used the three-step model, we do not have to be confined by semantic or even conceptual details of that model in the discussion here. The central argument here is what step in the whole process of origin and evolution of RNA editing arose in a neutral way. In our view, the only neutral aspect involves the mutations that contributed to the origin of RNA editing activity, i.e., the nature of mutational change is neutral. Fixation of these mutations and thus the RNA editing activity, as well as editable sites and mutations at those sites (only C <-> T changes, not any others) were all under selection*.

*The other important point we want to add here is that while the nature of mutational changes leading to the origin of RNA editing activity is neutral, the genomic environment in which these mutations arose is a history-constrained one (most of that history has been under selection). To put it explicitly, these mutations must represent a subset of all possible mutations that could possibly occur and the subset is shaped by the history of the genome, which is a selective one*.

*Finally, we actually disagree with the statement "a primary tenet in evolutionary theory is that evolution cannot be and is not anticipatory". In our view, evolution of life on earth is proceeding in a way that is shaped by the constant inflow of solar energy into the life system, and with sufficient knowledge, evolution of life on this planet is anticipatory*.

Another issue that is not generally considered is the effect that editing may have on the efficiency of translation of an edited mRNA, not just its translatability *per se *or the properties of the final protein product. In the case of plant mitochondria, the tRNA population is a mix of 'native' mtDNA-encoded tRNAs inherited vertically from a bacterial ancestor, 'chloroplast-like' tRNAs expressed from pieces of chloroplast genome that have become incorporated into the mitochondrial genome, and imported (nucleus-encoded cytosolic) tRNAs. Because the mix of these three types differs in diverse plants, it seems to me that editing might play some role in re-tailoring a message so that it is more efficiently translated by the particular mitochondrial tRNA population in a given plant species, an idea explicitly outlined in the case of dinoflagellate mRNA editing [Lin S, Zhang H, Spencer DF, Norman JE, Gray MW. Widespread and extensive editing of mitochondrial mRNAS in dinoflagellates. *J Mol Biol*. 2002. Jul 19;320(4):727-39].

#### Authors' response

*This is an interesting fact to consider. However, only seed plants have a mix of 'native' mtDNA-encoded tRNAs inherited vertically from a bacterial ancestor, 'chloroplast-like' tRNAs expressed from pieces of chloroplast genome that have become incorporated into the mitochondrial genome, and imported (nucleus-encoded cytosolic) tRNAs. The mitochondrial genomes of bryophytes lack any tRNA genes from the chloroplast genome, yet RNA editing almost certainly already occurs in the moss Physcomitrella patens and the hornwort Megaceros aenigmaticus mitochondria (Li, L., B. Wang, Y. Liu, & Y.-L. Qiu. 2008. The complete mitochondrial genome sequence of the hornwort Megaceros aenigmaticus reveals a mixed mode of conservative yet dynamic evolution in early land plant mitochondrial genomes. J. Mol. Evol. submitted). Whether the mixed population of tRNAs has anything to do with RNA editing remains to be investigated, but sufficient data are not available at present*.

Just as Covello and I considered that the RNA editing machinery (whatever it is) would have emerged under neutral conditions, we imagined that editable sites would have emerged in the same way, perhaps as a result of the GC-drive I mentioned earlier. In our model, we defined "editable positions or sites at the level of DNA not only as sites that are capable of being edited (the conventional meaning of editable), but also those that already correspond to the edited sequence. In the case of plant organelles in which C→U editing occurs, editable sites can be recognized at the RNA level by the editing machinery and contain either a T or a C in the DNA at the position that is actually edited. The mutations in Step 2 would be between Ts and Cs at these editable positions in the DNA." Thus, not every C position in a sequence is an editable site, only those that can be recognized by the RNA machinery. Importantly, we also argued that "Step 1, the appearance of RNA editing activity, has the very important effect of making these transitions neutral." We actually did say that it "is possible to argue for a selective advantage of RNA editing, perhaps as a result of the different nucleotide composition of DNA and RNA (i.e. higher GC content in kinetoplastid and plant organellar DNA)", although admittedly we went on to say that "we do not think this suggestion can easily be supported." Given the data now available and considering some of the arguments Jobson and Qiu make, I would be more inclined now to accept that possibility, although again I would point out that the distinction between 'origin' and 'maintenance' becomes quickly blurred in these models.

In regard to the authors' statement, "If mutations at editable sites were fixed through genetic drift, one would expect their distribution patterns to be completely random when examined against codon positions, types of codons, and kinds of genes", I would argue that's not necessarily the case if, in fact, one accepts that (i) editable sites, by definition, are non-random because they have to be capable of being recognized by the editing machinery (otherwise non-editable T→C mutations would quickly be eliminated by purifying selection), and (ii) the three codon positions are not equally C-rich (in angiosperms, at least, the 3rd codon position is ~40% T).

#### Authors' response

*Regarding point (i): Once the structural and functional aspects of the editing machinery are solved we will be in a better position to answer this question. Does the machinery have to recognize a single site? If it utilizes a sequence motif, then the conversion will depend on the nucleotide composition of the motif*.

### Reviewers report 2: Kirsten Krause, Department of Biology, University of Tromsø. Solicited by Martin Lercher (lercher@cs.uni-duesseldorf.de)

Jobson and Qiu present an analysis of RNA editing sites in two chloroplast genomes and, three mitochondrial genomes of land plants. With this, they aimed at improving our understanding of how the two editing reactions (C to U and U to C conversion) that exist in the plant organellar genomes could have evolved.

I have two major (I. and II.) and two minor problems (III. and IV.) with the way this work is presented and interpreted:

I. The authors present data that were by and large known before, but they offer a different interpretation of these data. The focus of their manuscript is, therefore, more towards presenting and justifying a new hypothesis that may help to unravel the mystery of how RNA editing in plant organellar genomes evolved, than towards a gain of actual and reproducible knowledge. Unfortunately, the authors were quite biased in the presentation of their arguments. They amply refer to the important paper of Covello and Gray of 1993 on the evolution of RNA editing while the thorough and much more recently published broad scale analysis by Tillich et al. (2006) is almost unmentioned. If this manuscript is to present a valuable reference for future research, the authors should take a broader approach in their discussion and name all the pros and cons of the different views on a still undecided question. In my opinion, the study by Tillich et al. (2006) has taken such a broad approach already with an investigation of the plastid genomes of several taxa that were similar or even identical to those used in the present study, including also a discussion on plant mitochondrial genomes. The authors should mention this more explicitly here and point out where their own analysis deviates from this previous work.

#### Authors' response

*We agree that the Tillich et al*., [26]* study is comprehensive and thought provoking, and we have discussed and cited the work in the current paper. However, in our view the 'preferred sequence context' pattern is most likely an artifact of: 1) the observed higher frequency of TTA (UUA) codons in organellar genomes of plants (~X3 that of TTG in Anthoceros cp CDS), in combination with 2) the fact that Leu is very common in transmembrane regions, and 3) UCA→UUA is one of the most common edits. Therefore, you would expect a high probability of an A or T up or down stream of an edit site. Whether or not the thus-far unknown editing machinery structure requires such a recognition pattern obviously cannot yet be determined*.

II. The authors state quite correctly in the introduction that seed plants have experienced a loss of editing sites in their plastids compared to the more primitive ferns and hornworts. This is an important fact that should be included in the discussion about the evolution of RNA editing because it shows that it is an ongoing mechanism and not a thing of the past. Unfortunately, the authors excluded the plastid genomes of seed plants from their analysis and also largely from their discussion. It might be worthwhile to reconsider this decision and elaborate more on how the fact that RNA editing of many but not all sites seems to have been lost, fits in with their interpretation. What made some sites apparently prone to mutation at the DNA level whereas other sites still depend on a change at the RNA level?

Alternatively, the authors should drop their claim of presenting a broad scale analysis and should clearly restrict their arguments to those organelles and taxa they analyzed.

#### Authors' response

*The RNA edited codon frequency pattern we observed (e.g., often UUR) in chloroplasts of Anthoceros and Adiantum, and mitochondria of angiosperms (involving hundreds of sites across 29–58 genes), is similar to that found across the ~15–44 editing sites (most in just a few genes) in seed plant chloroplast genomes. The several studies we have cited contain tables of the edited codons. We have included in the text a statement explaining that these codon data fit our model (e.g., mostly UUR), and we refer the reader to the codon tables in the relevant literature*.

III. Furthermore, it has been suggested by other authors that there seems to be an inverse correlation between mutation rates and RNA editing frequency and that,....

#### Authors' response

*An intriguing observation. To test this hypothesis we performed correlation analyses of editing frequency versus gene specific rates of evolution across nonsynonymous (dN) and synonymous (dS) sites *(Fig. 6), *and found an inverse relationship for dN, but not dS. So it seems that the suggested inverse relationship is associated more with selection constraint on the gene than with the overall mutation rate. Such an analysis can be confounded by the editing sites themselves. For this reason we removed the editing effect from the sequences, and sampled across a broad range of taxa*.

...therefore, RNA editing originated as a mechanism to complement the low mutation rates in the organellar genomes.

#### Authors' response

*This is an interesting idea; however, we wonder why it would be the case? The genome does not 'know' that it has a low or high mutation rate. Unless there is, for example, some environmental change that places selection pressure on the genome to undergo changes in function, would you expect the genomic diversity to really matter if the required enzymes are still properly produced for Mt function? For the evolution and maintenance of RNA editing sites, it seems that changes in mutation rate only matter if mutation rate and negative selection are not in sync. This process would best be determined through population genetic studies that consider life history and/or ecological factors*.

In this context, recent data describing an even further reduction in RNA editing activity in the reduced plastid genomes of some parasitic plant species which exhibit a high rate of mutations in their plastid genome (Funk et al. 2007, BMC Plant Biology 7:45) would be well worth discussing.

#### Authors' response

*We thank the reviewer and have now cited the work of Funk et al. 2008. One would expect reduced RNA-editing activity in a genome that is in a state of functional degradation*.

IV. The argument that reaching a desired GC-content (or avoiding a presumed upper limit of % A+T) could have promoted the fixation of the genes encoding enzymes involved in RNA editing is very questionable as even in the most extensive cases editing activity concerns just a relatively tiny fraction of sites compared to the total amount of nucleotides in coding sequences and therefore cannot have altered the G+C content by more than a fraction of one percent.

#### Authors' response

*As clearly outlined in the preceding sentence of the text, we do not suggest that the editing sites themselves have altered the nucleotide frequency, or that editing evolved to attain "desired" nucleotide frequencies. Instead, we suggest that whole genome processes of nucleotide compositional re-organization during early land plant diversification (see *Fig. 7*) could be selectively unfavorable within and across most of the protein coding genes particularly regarding a loss of hydrophobicity in membrane-bound proteins. The editing machinery probably evolved to maintain protein structural fidelity in the face of the compositional re-organization mentioned above. The underlying process of nucleotide compositional re-organization could then occur in these genes, and the rest of the genome, dependent on levels of selective constraint (see Selaginella uncinata *Fig. 7). *As the nucleotide composition stabilized in seed plants *(Fig. 7), *the corresponding editing frequencies have lowered. We do not yet have the Mt genome-transcriptome for an early land plant (e.g., hornwort, fern). However, from single locus studies of Mt genes it seems likely that editing site frequencies in these early lineages will greatly exceed those found in Mt genomes of seed plants*.

### Reviewers report 3: Jeffery Mower, Center for Plant Science Innovation, University of Nebraska Lincoln. Solicited by David Ardell (dardell@ucmerced.edu)

This manuscript attempts to address questions regarding the origin and evolution of RNA editing. Specifically, the authors wish to explore whether selection played a more prominent role than has been previously appreciated. I have several questions and concerns regarding their conclusions which are outlined below.

1. The authors make the very strong assertion that the functional basis of RNA editing is to increase hydrophobicity. I'd like to point out some facts that are not consistent with this assertion:

a) For reverse edits, the most heavily edited codons (that do not fix stop codons) are those that cause large decreases in hydrophobicity (C>R and L>P). If their assertion is correct, you'd expect that reverse edits causing large decreases in hydrophobicity would be the least frequent, not the most frequent. Thus, although the function of forward editing may be to increase hydrophobicity, this cannot be the case for reverse editing because it does the exact opposite.

b) The authors claim on p. 14 that the abundance of S>L and P>L changes clearly supports the role of editing to increase hydrophobicity. However, why is it then that T>I changes are one of the least frequent types of edits? On the Kyte-Doolittle scale, T>I changes would cause a greater increase in hydrophobicity than would S>L changes. Increasing hydrophobicity cannot be the only driving factor.

#### Authors' response

*We do not suggest that the role of editing is 'to increase hydrophobicity', although without editing a change in hydrophobicity would be the 'effect'. Obviously maintenance of hydrophobicity is under strong selection in genomes containing mostly membrane-bound protein coding genes. Instead, we have investigated the cause, or rather, the genomic environment in which RNA editing may have arisen. In early land plants a pattern of nucleotide re-arrangement was taking place *(Fig. 7) *and editing probably evolved to compensate for such changes in nucleotide frequency – possibly driven by increased C<->T transitions*.

*If editing activity suddenly ceased, there would be an overall decrease in hydrophobicity (ignoring the effects of edited stop codons). That is not to say that a proportion of edit sites would not 'increase' hydrophobicity – and these may well be equally deleterious or disruptive. Our precise point is that editing restores amino acids with the 'correct' physico-chemical properties. In the genomes we examined, this pattern is most obvious regarding increased hydrophobicity after editing*.

c) Compositional analyses have shown that there is a strong preference for pyrimidine immediately upstream of edited sites and a mild preference for purine immediately downstream. This preferred sequence context of edit sites goes a long way in explaining the patterns observed in Fig. 1, and it provides a clear explanation for the abundance of S>L and P>L changes and dearth of T>I and A>V changes. I suspect that editing frequency would correlate better with sequence context than with hydrophobicity change.

#### Authors' response

*The 'preferred sequence context' should correlate well with editing frequency, as does the frequency of NUN codons *(see Additional file 1), *because they are basically the same phenomenon. In our view this does not explain anything more regarding the origin and maintenance of RNA editing than what we present in *Figures 4* and *5. *Please refer to our explanation in Reviewer 2 responses*.

2. I believe the authors have failed to capture all of the complexities in their discussion of editing's origin and evolution. a) When thinking about the evolution of editing there are three states that must be considered. For a forward edit site you could have 1) a genomically encoded T, 2) an edited C, or 3) an unedited C. Clearly, at these positions an edited C is preferable to an unedited C, which is the simple reason for the maintenance of this edited site by selection. But when discussing the fixation of a new edit site, you must compare the edited C state to the ancestral state of a genomic T. What is the selective benefit to going from a genomic T to an edited C? Clearly it cannot be some protein-level effect (such as hydrophobicity), because both states generate a U in the RNA leading to identical AAs.

#### Authors' response

*We have addressed this misunderstanding above. The protein level 'effect' is what would happen if editing suddenly ceased. The question of how sites are fixed as edit sites in the first instance is a question that requires population level studies. It is probable that during the divergence of land plants, lineages underwent periods of reduced selection pressure. The 'editing machinery' was probably a means of fixation of selectively constrained pyrimidines under these reduced selection periods. Once the machinery was established the edited sites could persist and be edited, persist and be unedited, or change back to the original pyrimidine (purified)*.

b) Elsewhere the authors argue that the non-random distribution of editing is evidence that selection was responsible for their fixation and is incompatible with a neutral fixation scenario. I disagree with this statement. Instead, I would contend that the present-day distribution results from neutral fixation coupled with differences in the selection pressure to maintain edit sites (eg, there are so few 3rd position edit sites because they are silent at the protein level so there is no selection to maintain them).

#### Authors' response

*Good points. However, the reviewer has left out an important point in the argument: Selection on the editing machinery to establish fixation in the first instance*.

3. I don't see the big conclusions to be drawn from Fig. 4 &5 and its associated text. The first observation is that "RNA editing sites occur more frequently in ancestrally T-rich sequences." This really isn't all that surprising as the authors also point out. I certainly don't find it necessary to see the massive figure 4 to believe this; a simple statement would suffice. The other observation is that "genes encoding membrane-bound proteins tend to have larger T – A distances." Isn't this simply because codons with T code for more hydrophobic AAs than codons with A, and membrane-bound proteins require more of these T-rich codons? For example, codons with T at the 2nd pos (FLIMV) are very hydrophobic, whereas codons with A at the 2nd pos (YHQNKDE) are not. Again, I don't see the necessity of the figure or the text in the manuscript.

#### Authors' response

*Regarding our observation; 'RNA editing sites occur more frequently in ancestrally T-rich sequences', the reviewer states "This really isn't all that surprising". The fact that it is not surprising does not mean it is not important regarding the genomic environment in which RNA editing evolved *– Figures 4 &5*were not only intended to demonstrate that a T-rich sequence is prone to more C mutations. If this were all we wanted to demonstrate, we would have presented the T composition against the editing frequency. The T-A distance [%(T - A)/(T+A)] represents the increase or decrease of T relative to the composition of A. We have described our reasons for its use in the paper. As the reviewer suggested, we have reduced the size of *Figures 4 &5.

p. 5, 2nd para: For chloroplasts, quite a few other species have been analyzed for editing, such as Phalaenopsis, Pisum, Solanum, Oryza and Saccharum.

#### Authors' response

*We have cited the Saccharum and Phalaenopsis papers. The Phalaeopsis paper contains a table of all known angiosperm chloroplast edit sites*.

On the other hand, only a small fraction of Zea mitochondrial genes has been evaluated for editing, so it should not be listed as complete.

#### Authors' response

*We have made this clear by including "partially" as suggested by the reviewer – "and partially for Zea mays *[42]."/p. 10, 2nd para: According to Grantham, the difference in index between Pro > Ser change is due to their "composition" (the third property that was measured), not to their polarity or volume. Thus, the statement that all of the changes listed in that paragraph involve a large change in hydrophobicity and molecular size is incorrect.

#### Authors' response

*According to the hydrophobicity scales below, hydrophobic differences between Pro and Ser residues constitute a decent shift in hydrophobicity*.

- Kyte-Doolittle hydrophobicity scale: Pro = -0.8, Ser = -1.6

- Hopp-Woods hydrophobicity scale: Pro= 0.0, Ser= 0.3

*In light of the Grantham *[47]* discrepancy we have included the citation for Kyte-Doolittle *[48]* to support our statement*.

Fig 3 – This figure is unnecessarily complex, making it very difficult to compare across plots. 1) Each plot should be at the same scale. 2) It would be much easier to compare if the bars for each AA were side-by-side in the same chart. 3) If the figure is attempting to contrast AA content between membrane-bound and information processing proteins, why not simply split genes into those two categories rather than into six? 4) The authors need to state that the amino acids are ordered by the Kyte-Doolittle hydropathy scale, if this is indeed how they were ordered. p. 13, 2nd para: The authors state that Leu is the most frequent "in all classes of proteins except those involved in information processing..." In contrast to this statement, Leu also ranks highest for *Anthoceros *ribosomal subunits + polymerases (Fig 3A plot v).

#### Authors' response

*The sentence now reads: "Leu consistently ranked the highest among all 20 amino acids...". Also, we have simplified *Figure 3* as suggested by the reviewer, with combined 'membrane-bound' and 'soluble' categories for both mitochondria and chloroplasts. We have also stated in the *Fig 3* legend that residues are ordered according to the Kyte-Doolittle hydrophobicity scale*.

## Supplementary Material

Additional file 1 Correlation analyses of editing frequency versus nucleotide composition. Table of correlations for forward (C→U) and reverse (U→C) editing frequency at 1^st^, 2^nd^, 3^rd^, and total codon positions versus combined composition of aliphatic NUN codons (I, V, L), CRR (Q+R) codons, and ratios of compositional distance between nucleotide pairs T-A, and C-G, at 1^st^, 2^nd^, 3^rd^, and total codon positions. Analyses were performed across 68 chloroplast protein-coding genes from *Anthoceros formosae *(*Af*), and *Adiantum capillus-veneris *(*Acv*), and 30 mitochondrial protein-coding genes from *Beta vulgaris *(*Bv*). Significant two-tailed Pearson correlations are signified by * at the 0.05 level, and ** at the 0.01 level. 'a' = cannot be computed because at least one of the variables is constant. 'na' = not applicable. Relevant data were used in Figures 4 and 5. Click here for file

Additional file 2 Evolutionary analysis of total editing frequency across genes. Input trees used for chloroplast and mitochondrial gene specific *dN *and *dS *analyses (Figure 6) are presented. Chloroplast and mitochondrial genes used in gene specific *dN *and *dS *analyses are listed according to each analyzed taxon. Data include the *dN*/*dS *ratio, *dN, dS*, transition/tranversion ratio (Ti/Tv), alignment (matrix) length, and total editing frequency for each gene. Information is divided into mitochondrial and chloroplast data-sets. Soluble proteins are shown in italics. Click here for file

Additional file 3 Taxonomic and genome information. The data provide a full description of taxon names for those abbreviated in Figure 7, along with associated GenBank numbers. Genomes used to construct input trees for gene specific *dN *and dS analyses (Figure 6, Additional file 2) are shown in bold. Click here for file
