# Supplementary material for: Did RNA editing in plant organellar genomes originate under natural selection or through genetic drift?
Source: Biol Direct. 2008 Oct 21;3:43. doi: 10.1186/1745-6150-3-43 (PMC2584032; doi:10.1186/1745-6150-3-43)
Supplement: Additional file 1 — Correlation analyses of editing frequency versus nucleotide composition. Table of correlations for forward (C→U) and reverse (U→C) editing frequency at 1st, 2nd, 3rd, and total codon positions versus combined composition of aliphatic NUN codons (I, V, L), CRR (Q+R) codons, and ratios of compositional distance between nucleotide pairs T-A, and C-G, at 1st, 2nd, 3rd, and total codon positions. Analyses were performed across 68 chloroplast protein-coding genes from Anthoceros formosae (Af), and Adiantum capillus-veneris (Acv), and 30 mitochondrial protein-coding genes from Beta vulgaris (Bv). Significant two-tailed Pearson correlations are signified by * at the 0.05 level, and ** at the 0.01 level. 'a' = cannot be computed because at least one of the variables is constant. 'na' = not applicable. Relevant data were used in Figures 4 and 5. [file 1745-6150-3-43-S1.rtf]

	

                  Edit Freq.	CU
2	CU
3	CU
tot 	UC
1	UC
2	UC
3	UC
tot	Aliphatic
(I, V, L)	CRR
(Q, R)	C-G
 1	C-G
 2	C-G
 3	C-G
tot	T-A
1	T-A
2	T-A
3	T-A
tot	
Af
(N=68)	CU 1	.336**
(.005)	-.089
(.469)	.542**
(.000)	.228
(.062)	.525**
(.000)	-.049
(.690)	.431**
(.000)	.049
(.690)	.040
(.747)	-.167
(.173)	-.059
(.630)	-.217
(.076)	-.245*
(.044)	.179
(.144)	.102
(.406)	-.196
(.110)	.045
(.716)	

	CU 2		-.032
(.798)	.936**
(.000)	.303*
(.012)	.555**
(.000)	-.077
(.533)	.502**
(.000)	.327**
(.006)	-.188
(.125)	-.160
(.191)	.135
(.272)	-.078
(.528)	-.067
(.584)	.250*
(.040)	.341**
(.001)	.090
(.464)	.321**
(.008)	

	CU 3			.206
(.092)	-.128
(.298)	.218
(.074)	.660**
(.000)	.225
(.066)	.133
(.279)	-.244*
(.045)	-.080
(.515)	.112
(.365)	-.090
(.464)	-.010
(.933)	.069
(.574)	.170
(.165)	-.072
(.557)	.087
(.479)	
	CU tot				.290*
(.017)	.675**
(.000)	.091
(.459)	.606**
(.000)	.327**
(.006)	-.211
(.084)	-.204
(.096)	.125
(.310)	-.151
(.219)	-.131
(.288)	.280*
(.021)	.397**
(.001)	.003
(.978)	.308*
(.011)	

	UC 1					.268*
(.027)	-.094
(.446)	.731**
(.000)	-.239
(.050)	.340**
(.005)	-.024
(.849)	-.380**
(.001)	-.291*
(.016)	-.477**
(.000)	-.078
(.527)	-.179
(.143)	-.160
(.194)	-.171
(.164)	

	UC 2						.226
(.064)	.789**
(.000)	-.033
(.791)	.022
(.860)	-.233
(.056)	.044
(.723)	-.335**
(.005)	-.255*
(.036)	.144
(.242)	.133
(.278)	-.133
(.281)	.076
(.539)	

	UC 3							.336**
.005	-.038
(.760)	-.045
(.716)	-.016
(.897)	.125
(.311)	-.155
(.206)	.000
(.999)	-.133
(.281)	-.018
(.887)	-.146
(.234)	-.110
(.371)	
	UC tot								-.137
(.264)	.187
(.127)	-.129
(.295)	-.196
(.110)	-.413**
(.000)	-.448**
(.000)	.013
(.919)	.004
(.973)	-.198
(.106)	-.061
(.620)	
Acv
(N=68)	CU 1	.572**
(.000)	.035
(.775)	.724**
(.000)	.075
(.546)	-.072
(.562)	a
-	.059
(.635)	.217
(.076)	-.109
(.377)	-.187
(.127)	.113
(.360)	-.165
(.179)	-.044
(.720)	.195
(.111)	.233
(.056)	.005
(.968)	.212
(.083)	
	CU 2		.121
(.327)	.969**
(.000)	-.050
(.685)	.214
(.080)	.a
-	-.004
(.975)	.338**
(.005)	-.085
(.490)	-.132
(.284)	.223
(.068)	-.193
(.115)	-.027
(.827)	.149
(.224)	.312**
(.002)	.013
(.917)	.261*
(.032)	
	CU 3			.193
(.114)	-.017
(.893)	-.043
(.728)	.a
-	-.026
(.836)	.001
(.991)	-.021
(867)	.036
(.768)	-.076
(.540)	.071
(.566)	.039
(.753)	-.002
(.987)	.017
(.892)	.141
(.252)	.056
(.651)	
	CU tot				-.033
(.790)	.139
(.259)	.a
-	-.003
(.981)	.323**
(.007)	-.090
(.466)	-.153
(.212)	.208
(.089)	-.199
(.103)	-.028
(.822)	.169
(.169)	.355**
(.003)	.038
(.760)	.272*
(.025)	
	UC 1					-.064
(.604)	.a
-	.977**
(.000)	-.208
(.089)	.258*
(.034)	-.120
(.329)	-.310*
(.010)	-.241*
(.048)	-.375**
(.002)	-.379**
(.001)	-.243*
(.046)	-.226
(.064)	-.375**
(.002)	
	UC 2						a
	.150
(.221)	.130
(.290)	-.187
(.126)	-.133
(.280)	.168
(.172)	.142
(.248)	.094
(.443)	.091
(.460)	.137
(.265)	.003
(.980)	.116
(.348)	
	UC 3							a	a	a	a	a	a	a	a	a	a	a	
	UC tot								-.178
(.147)	.216
(.147)	-.148
(.230)	-.271*
(.025)	-.209
(.088)	-.352**
(.003)	-.356**
(.003)	-.212
(.083)	-.223
(.068)	-.347**
(.004)	
Bv
(N=30)	CU 1	.755**
(.000)	.421*
(.023)	.899**
(.000)	na
-	na
-	na
-	na
-	.353
(.061)	-.249
(.192)	.308
(.104)	-.123
(.525)	-.132
(.496)	.084
(.664)	.592**
(.005)	.272
(.153)	.319
(.092)	.391*
(.036)	
	CU 2		.379*
(.043)	.953**
(.000)	.na
-	.na
-	.na
-	.na
-	.453*
(.014)	-.286
(.133)	-.044
(.819)	.136
(.482)	-.223
(.244)	-.075
(.700)	.418*
(.024)	.491*
(.043)	.249
(.192)	.389*
(.037)	
	CU 3			.535**
(.003)	.na
-	.na
-	.na
-	.na
-	-.035
(.859)	.012
(.952)	.148
(.443)	.039
(.839)	-.040
(.837)	.131
(.499)	.275
(.149)	-.043
(.825)	.069
(.724)	.095
(.623)	
	CU tot				.na
-	.na
-	.na
-	.na
-	.406*
(.029)	-.267
(.161)	.109
(.573)	.044
(.822)	-.192
(.317)	.004
(.982)	.485**
(.008)	.328
(.082)	.283
(.137)	.399*
(.032)	
